# Supplementary material for: A Mini Chalk Talk Workshop for Fourth-Year Medical Students: Facilitating the Transition From Student to Resident Educator
Source: MedEdPORTAL. 2024 Jun 25;20:11404. doi: 10.15766/mep_2374-8265.11404 (PMC11219125; doi:10.15766/mep_2374-8265.11404)
Supplement: Supplementary file 1 — Presurvey Questions.docxHow to Prepare an Effective Mini Chalk Talk Video.mp4Mini Chalk Talk Tip Sheet.docxMini Chalk Talk Observation Form.docxMini Chalk Talk Preparation Worksheet.docxFacilitator Email.docxSample Mini Chalk Talk.mp4Postsurvey Questions.docx [file mep_2374-8265.11404-s001.zip › A. Presurvey Questions.docx]

**Mini-Chalk Talk Workshop Pre-Survey**

*This survey should be sent to students at the beginning of the course to indicate interest in the workshop.*

What is your name?

[short answer]

What specialty are you going into?

[short answer]

How well can you describe strategies for effectively delivering a mini-chalk talk?

Not at all well

Slightly

Moderately well

Quite well

Extremely well

How confident do you feel in your ability to give an effective mini-chalk talk?

Not at all confident

Slightly confident

Moderately confident

Quite confident

Extremely confident

How many relevant mini-chalk talks relevant to your internship specialty do you currently have in your repertoire?

0

1

2-3

4-5

6 or more
