# Supplementary material for: A Mini Chalk Talk Workshop for Fourth-Year Medical Students: Facilitating the Transition From Student to Resident Educator
Source: MedEdPORTAL. 2024 Jun 25;20:11404. doi: 10.15766/mep_2374-8265.11404 (PMC11219125; doi:10.15766/mep_2374-8265.11404)
Supplement: Supplementary file 1 — Presurvey Questions.docxHow to Prepare an Effective Mini Chalk Talk Video.mp4Mini Chalk Talk Tip Sheet.docxMini Chalk Talk Observation Form.docxMini Chalk Talk Preparation Worksheet.docxFacilitator Email.docxSample Mini Chalk Talk.mp4Postsurvey Questions.docx [file mep_2374-8265.11404-s001.zip › H. Postsurvey Questions.docx]

**Mini-Chalk Talk Workshop Post-Survey**

*This survey should be administered immediately after the MCT session.*

How well can you describe strategies for effectively delivering a mini-chalk talk?

Not at all well

Slightly

Moderately well

Quite well

Extremely well

How confident do you feel in your ability to give an effective mini-chalk talk?

Not at all confident

Slightly confident

Moderately confident

Quite confident

Extremely confident

How many relevant mini-chalk talks relevant to your internship specialty do you currently have in your repertoire?

0

1

2-3

4-5

6 or more

How educational was today's workshop with respect to learning clinical information?

Not at all

Slightly

Moderately

Quite

Very

How educational was today’s workshop with respect to developing your teaching skills?

Not at all

Slightly

Moderately

Quite

Very

What would you modify about today’s workshop?

[free response]

What was effective about today’s workshop?

[free response]
