# Supplementary material for: A Mini Chalk Talk Workshop for Fourth-Year Medical Students: Facilitating the Transition From Student to Resident Educator
Source: MedEdPORTAL. 2024 Jun 25;20:11404. doi: 10.15766/mep_2374-8265.11404 (PMC11219125; doi:10.15766/mep_2374-8265.11404)
Supplement: Supplementary file 1 — Presurvey Questions.docxHow to Prepare an Effective Mini Chalk Talk Video.mp4Mini Chalk Talk Tip Sheet.docxMini Chalk Talk Observation Form.docxMini Chalk Talk Preparation Worksheet.docxFacilitator Email.docxSample Mini Chalk Talk.mp4Postsurvey Questions.docx [file mep_2374-8265.11404-s001.zip › C. Mini Chalk Talk Tip Sheet.docx]

**
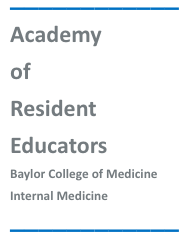
Chalk Talk Tip Sheet**

*This sheet should be sent to interested students and facilitators prior*

*to the session.*

**PREPARATION**

*“Draw up your bow and arrow”*


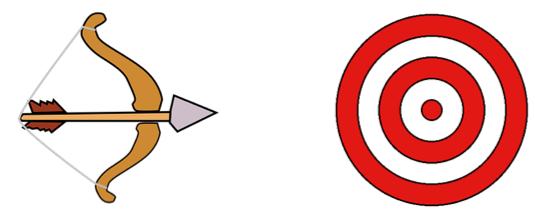


1**. Select a target audience**

- Selecting a group will help you choose the most appropriate topic and decide how detailed to make your talk
- Suggestions: medical students, sub-intern, interns, or all of the above

1. **Select a narrow topic**

- It’s okay to teach to your strengths!
- Try to make the topic as specific as possible.
- e.g. instead of choosing “diabetes,” try diabetes chronic care, DKA diagnosis, DKA management, or DKA versus HHNK
- Select a topic that is relevant to your audience. Medical students are more likely to benefit from discussions about diagnosis or pathophysiology whereas interns are more likely to benefit from talks about diagnosis and management.
- Understand the appropriate depth/scope of the topic for your specific audience
- e.g. reference curricular documents or commonly used resources like *Step Up to Medicine* for core medicine students

| **Topics that work well for chalk talks** | **Topics that do NOT work well for chalk talks** |
| --- | --- |
| - Fundamental concepts needed to understand more complex ideas in a subject (e.g. talk on flow–volume loops) - Concepts that learners tend to find difficult or have identifiable misconceptions about (e.g. distinction between metabolic and respiratory acidosis) - Concepts that highlight causal pathways or relationships among topics (e.g. comparison of metabolic pathways, pathophysiology of obstructive lung diseases) - Topics that interest your learners | - Facts that can easily be referenced in textbooks or do not have visual representations (e.g. epidemiology and specific management recommendations for a disease) - Topics that are esoteric or not of primary relevance to learners (e.g. rare disease sub-types) |

1. **Choose your teaching objectives**

- Give your learners 2-3 learning objectives to ensure what they remember are the most important points of your talk
- Each goal should complete the sentence “The learner will be able to…”
- For example, for atrial fibrillation treatment:
- Recall 2 classes of medications used as first-line agents to rate-control AF in a normotensive patient.
- Recall 2 classes of medications used to rate-control AF in hypotensive patients.
- Use risk calculators to determine indication for anticoagulation

1. **Create your content**

- Plan the organization of your board
- Use advanced organizers
- e.g. dyspnea pyramid for management of dyspnea
- Draw flow charts
- e.g. sequence for management of thyroid nodules
- Incorporate mnemonics
- For structure of talk, e.g. **AEIOU** mnemonic (**A**cidosis, **E**lectrolytes, **I**ngestion, **O**verload, **U**remia) to structure a talk on acute indications for dialysis
- For small points, e.g. in talk on management of pneumonia, remembering levofloxacin is typically used in pulmonary infections because **L**evofloxacin and **L**ung both start with **L**

**Source**: OnlineMedEd.com

**PRESENTATION**

*“Fire the arrow”*

*
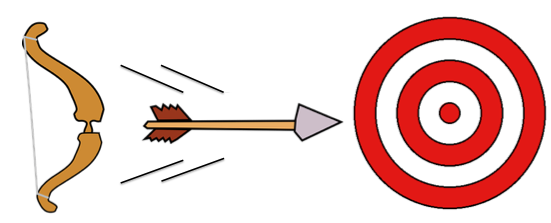
*

1. **Start with a hook**

- Set a scenario
- e.g. painting a picture for a sub-I of getting called on night float for chest pain prior to a talk on developing a differential for chest pain
- Appeal to relevance in patient care and thus frequency in test questions
- Consider catering to learners’ interests
- e.g. engaging a learner interested in orthopedic surgery in a talk on antibiotic selection in osteomyelitis

1. **State your objectives**

- “The 3 things I want you to remember after this talk are…”

1. **Teach your content!**

| **Teaching strategies** | **Engagement strategies** |
| --- | --- |
| 1. Repetition of important concepts 2. Summarization of discrete components of the talk 3. Enumeration: “There are three medications to remember for this concept… one… two… three” | 1. Safe learning environment: positive reinforcement encourages participation, e.g. “Great question…” “I like the way you’re thinking…” 2. Check for understanding: “what questions do you have?” “I think this part can sometimes be confusing, do you have any questions?” 3. Use of space: organization of board, body positioning, eye contact, legible handwriting |

1. **Conclusion**

- At the end, review your goals and reinforce correcting any misconceptions
- Don’t rush! It is okay not to finish all of your material. It is more important to be attuned to your audience’s needs.

**EVALUATION**

*“Check to see where your arrow landed”*


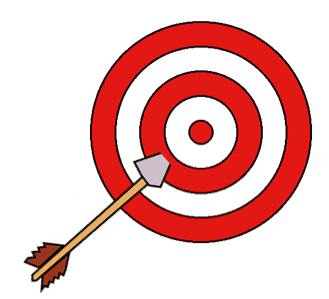


**Self-reflection**

Which parts of the talk went well? Were there areas that you felt confused your learners? Were your learners engaged throughout the talk?

**Feedback**

You can also ask others to observe you teaching and request feedback based on the chalk talk checklist.

- Content
- Didactic structure
- Teaching strategies
- Board use
- Time management

*All images contained in this document are author-owned but not copyrighted.*
