# Supplementary material for: A Mini Chalk Talk Workshop for Fourth-Year Medical Students: Facilitating the Transition From Student to Resident Educator
Source: MedEdPORTAL. 2024 Jun 25;20:11404. doi: 10.15766/mep_2374-8265.11404 (PMC11219125; doi:10.15766/mep_2374-8265.11404)
Supplement: Supplementary file 1 — Presurvey Questions.docxHow to Prepare an Effective Mini Chalk Talk Video.mp4Mini Chalk Talk Tip Sheet.docxMini Chalk Talk Observation Form.docxMini Chalk Talk Preparation Worksheet.docxFacilitator Email.docxSample Mini Chalk Talk.mp4Postsurvey Questions.docx [file mep_2374-8265.11404-s001.zip › D. Mini Chalk Talk Observation Form.docx]

**
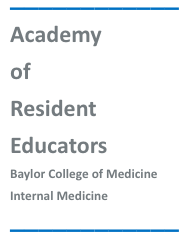
Mini-Chalk Talk Observation Form**

*This sheet should be sent to interested students and facilitators prior*

*to the session and should be used by the facilitator during the session to*

*facilitate feedback.*

**Topic:**

**Observer name:**

**Content:**

*Comments:*

❑ Appropriate topic selection

❑ Depth tailored to level of audience

❑ Use of advanced organizers, flow charts, mnemonics, etc.

*Comments:*

**Didactic structure:**

❑ Hook

❑ Clear teaching objectives

❑ Use of summary

*Comments:*

**Teaching strategies:**

❑ Check for understanding

❑ Create positive learning environment

❑ Repetition, summarization, enumeration

❑ Other: ____________________

*Comments:*

**Board use:**

❑ Space used effectively

❑ Legible handwriting

❑ Use of colors, pictures, diagrams

**Other:**

*Comments:*

❑ Length <6 minutes

❑ Humor
