# Supplementary material for: A Mini Chalk Talk Workshop for Fourth-Year Medical Students: Facilitating the Transition From Student to Resident Educator
Source: MedEdPORTAL. 2024 Jun 25;20:11404. doi: 10.15766/mep_2374-8265.11404 (PMC11219125; doi:10.15766/mep_2374-8265.11404)
Supplement: Supplementary file 1 — Presurvey Questions.docxHow to Prepare an Effective Mini Chalk Talk Video.mp4Mini Chalk Talk Tip Sheet.docxMini Chalk Talk Observation Form.docxMini Chalk Talk Preparation Worksheet.docxFacilitator Email.docxSample Mini Chalk Talk.mp4Postsurvey Questions.docx [file mep_2374-8265.11404-s001.zip › E. Mini Chalk Talk Preparation Worksheet.docx]

**
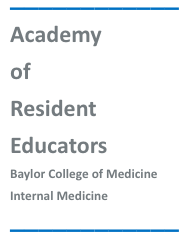
Mini-Chalk Talk Worksheet**

*This worksheet should be sent to students and facilitators prior to*

*the session.*

**Step 1**: Select your audience

**Step 2**: Select your topic

*Tip: Remember to make it narrow*

**Step 3**: Create 2-3 teaching objectives

*Tip: Each objective should complete the sentence “The learner will be able to…”*

1. _______________________________________________________________________
2. _______________________________________________________________________
3. _______________________________________________________________________

**Step 4**: Determine the content you will include

**Step 5**: Organize the content

*Tip: Use advanced organizers, flow charts, mnemonics, etc.*
