# Supplementary material for: A Mini Chalk Talk Workshop for Fourth-Year Medical Students: Facilitating the Transition From Student to Resident Educator
Source: MedEdPORTAL. 2024 Jun 25;20:11404. doi: 10.15766/mep_2374-8265.11404 (PMC11219125; doi:10.15766/mep_2374-8265.11404)
Supplement: Supplementary file 1 — Presurvey Questions.docxHow to Prepare an Effective Mini Chalk Talk Video.mp4Mini Chalk Talk Tip Sheet.docxMini Chalk Talk Observation Form.docxMini Chalk Talk Preparation Worksheet.docxFacilitator Email.docxSample Mini Chalk Talk.mp4Postsurvey Questions.docx [file mep_2374-8265.11404-s001.zip › F. Facilitator Email.docx]

Dear Mini-Chalk Talk Workshop facilitators!

This email will serve as your guide to facilitating the upcoming session where small groups of fourth-year medical students will be delivering mini-chalk talks to their peers and receiving some feedback on their teaching. Please read closely.

About a week ago, the students were asked to watch the attached 7-min video (which you should also watch) [Appendix B], choose a topic for their talk, and consider the format in which they would deliver their talks. They were also provided with the observation form which you should use to provide feedback on their talks (also attached) [Appendix D].

In the small group sessions tomorrow, you should take a couple minutes to have everyone introduce themselves, share their future career paths/goals, and include something personal about themselves (e.g. favorite show recently) to build some rapport. Subsequently, you will dive into the following sequence for each student/talk, which should take about 10-15 minutes per student/talk.

1. Student delivers their prepared talk
   1. Facilitator fills out mini-chalk talk observation form (attached) [Appendix D] while watching
2. Free-flowing feedback conversation
   1. Fellow students share what they thought was done well
   2. Fellow students share what student can consider modifying in future talks
   3. Facilitator adds any additional comments if needed
3. Fellow students name one take-home point from the talk (if not solicited already as part of the talk)
4. Facilitator finalizes observation form and emails to that student

After the session, please:

1. Make sure you have emailed all the observation forms to the students if were not able to keep up live
2. Email the session organizers designating a single talk which you thought was the best one in your group

Here are your group/student assignment if you’re curious. [Link to spreadsheet provided].

Thank you so much for your time

Best,

Mini-Chalk Talk Workshop Organizers
