# Supplementary material for: A Mini Chalk Talk Workshop for Fourth-Year Medical Students: Facilitating the Transition From Student to Resident Educator
Source: MedEdPORTAL. 2024 Jun 25;20:11404. doi: 10.15766/mep_2374-8265.11404 (PMC11219125; doi:10.15766/mep_2374-8265.11404)
Supplement: Supplementary file 1 — Presurvey Questions.docxHow to Prepare an Effective Mini Chalk Talk Video.mp4Mini Chalk Talk Tip Sheet.docxMini Chalk Talk Observation Form.docxMini Chalk Talk Preparation Worksheet.docxFacilitator Email.docxSample Mini Chalk Talk.mp4Postsurvey Questions.docx [file mep_2374-8265.11404-s001.zip › _Educational Summary Report_11404.pdf]

MedEdPORTAL® The AAMC Journal of Teaching and Learning Resources

Original Publication Open Access

A Mini Chalk Talk Workshop for Fourth-Year Medical Students: Facilitating the Transition From Student to Resident Educator

Trevor Jamison, MD*, Zaven Sargsyan, MD, Uma Ayyala, MD, Stephanie Sherman, MD, Holland Kaplan, MD

*Corresponding author: trevor.jamison@bcm.edu

Abstract

Introduction: There is increasing recognition that incoming interns benefit from formal training in teaching skills during UME. Many medical schools have capstone courses well suited for teacher-training content. Mini chalk talks (MCTs) are a common clinical teaching modality requiring a variety of teaching skills. We developed a session for our institution's capstone course in which students prepared and delivered MCTs. **Methods:** The voluntary flipped classroom session was offered virtually in 2021 and in person in 2022. Before the session, students reviewed materials on creating effective MCTs and developed and practiced their own MCT. During the 90-minute session, students presented their MCT to a group of students in the same or similar future specialties and received feedback from their peers and a facilitator. **Results:** Twenty-six percent of graduating students (95 of 370) in 16 specialties participated. Students had a statistically significant increase in confidence delivering effective MCTs ($p < .01$). On a 5-point Likert scale (1 = *did not learn,* 5 = *a great amount*), students' mean ratings of clinical knowledge and teaching skills gained from the session were 4.4 and 4.5, respectively. Qualitative feedback highlighted the benefits of receiving feedback on teaching (31 of 77 respondents, 40%), practicing teaching skills (21 of 77, 27%), and experiencing other students' MCTs (13 of 77, 17%). **Discussion:** Our MCT session provides a versatile, resource-efficient method of supporting students in transitioning to the role of resident educators. It also offers them an opportunity to receive valuable feedback on their teaching in a low-stakes environment.

Keywords
Chalk Talk, Internship Preparation, Capstone Course, Transition Course, Medical Student as Teacher, Flipped Classroom, Residency Preparation, Virtual Learning

Educational Objectives

By the end of this activity, learners will be able to:

1. Deliver a mini chalk talk (MCT) effectively.
2. Summarize the key elements required to effectively prepare and deliver an MCT.
3. Deliver and receive feedback on an MCT.

Introduction

Teaching is a fundamental clinical skill used by residents and practicing physicians, impacting not only their interactions with learners but also their engagement with patients.[1] Early in their postgraduate careers, interns play a key role teaching medical students in the clinical setting. While interns identify with the role of being a teacher,[2] there has been increased recognition that they would benefit from earlier and more formal development of teaching skills.[3,4] To help interested students prepare for their role as resident educators, about half of United States medical schools have implemented dedicated medical student-as-teacher electives, which typically include a series of teaching workshops and opportunities for peer teaching of junior medical students.[5-13] While such courses have the potential to improve students' teaching abilities[10] and foster identification with the role of educator,[2,10] they require intense investment of resources and faculty time for implementation, which may not be feasible for all settings. The capstone courses already present in many medical schools' final-year curricula may provide a time- and resource-efficient avenue for delivering medical student-as-teacher content. Some medical schools' capstone courses already incorporate not only essential clinical content but

Citation:
Jamison T, Sargsyan Z, Ayyala U, Sherman S, Kaplan H. A mini chalk talk workshop for fourth-year medical students: facilitating the transition from student to resident educator. *MedEdPORTAL.* 2024;20:11404. https://doi.org/10.15766/mep_2374-8265.11404

Copyright © 2024 Jamison et al. This is an open-access publication distributed under the terms of the Creative Commons Attribution license.

also other elements of professional development, including the enhancement of teaching skills.[14,15]

Mini chalk talks (MCTs) are a common method of teaching in the clinical setting. Due to their concise nature, clinically applicable content, and active engagement of learners,[16-18] MCTs are an ideal deliverable for student-as-teacher sessions. While MCT workshops for residents have been described,[19,20] facilitating such a workshop during a capstone course for medical students represents a novel context. Preparing and delivering MCTs require students to learn and apply teaching techniques commonly covered in dedicated student-as-teacher programs.[5,8] MCT workshops can meet students' and educational programs' common goals, such as developing skills for clinical teaching and facilitating small-group learning. These sessions also enable students to begin to develop a portfolio of MCTs relevant to their specialty, further facilitating their role as teachers as they transition to residency.[17]

In 2021 and 2022, our institution's existing fourth-year medical student capstone course[21] incorporated an optional MCT session. Here, we describe the implementation and outcomes of this resource-efficient and versatile MCT workshop.

Methods

Curriculum Development

Our institution's required 2-week capstone course for graduating medical students included both clinical and nonclinical topics to prepare students for internship in various specialties. The course was structured to provide general education on relevant topics to all students, as well as tailored tracks for internal medicine, pediatrics, obstetrics/gynecology, surgery, and psychiatry. Topics such as overnight common calls had been successfully delivered using small-group, case-based learning with both faculty and resident facilitators, demonstrating the feasibility of this model.[21] In 2021, this capstone course was conducted virtually due to the COVID-19 pandemic; in 2022, the course was conducted as a hybrid virtual/in-person course with 80% of sessions in person. Thus, our MCT session was conducted virtually in 2021 and in person in 2022. In both years, the session was optional. Consistent with a flipped classroom format, students reviewed content on how to deliver effective MCTs and prepared their own MCTs prior to the session. The 90-minute allotted class time was then used to deliver their MCTs and receive feedback. The preparation materials, described below, were created by the authors. Their earlier versions had been used in similar workshops implemented for internal medicine residents interested in medical education. The materials and session design were adapted for a medical student audience by providing more explicit details on teaching strategies, expanding the MCT creation worksheet to offer additional guidance, and modifying the content to fit within the context of the capstone course.

Student Preparation and Group Assignment

Students were informed about the optional MCT module by the course director during the introductory session of the capstone course. During the first week of the course, students were emailed details about the MCT session, including an electronic sign-up form and presession survey (Appendix A). Based on responses to this form, students were organized into groups of four to six on the basis of specialty. If a specialty was represented by fewer than four students, it was combined with another commonly collaborating specialty (e.g., emergency medicine and obstetrics/gynecology). Interested students were sent a second email that asked them to review a 6-minute video (Appendix B [author-owned but not copyrighted]), a document summarizing the strategies covered in the video (Appendix C), an MCT evaluation rubric (Appendix D), and a worksheet to help them outline their teaching scripts (Appendix E). They also received a sign-up sheet to sign up for an MCT topic, with instructions to avoid duplicating topics within the same group. The video (Appendix B) served the dual purposes of providing students with a sample MCT and teaching strategies for preparing effective MCTs. To achieve this, the learning objectives of the video were narrower than and complementary to the overall workshop objectives, akin to how session objectives can map onto course objectives in larger learning experiences. Students who participated in the virtual module in 2021 were given options for a variety of formats to present their MCTs, including (a) recording a narrated video of them writing on paper, (b) recording a video at a chalkboard or whiteboard, (c) live or recorded use of Zoom's whiteboard function, or (d) live or recorded use of one to three animated PowerPoint slides. Students who participated in 2022 were instructed to design their MCTs in a traditional chalk talk format to be presented in person with a whiteboard. Participants were encouraged to aim for an MCT duration of less than 6 minutes.

Facilitators

Three weeks prior to the workshop, academic faculty, fellows, and senior residents from various specialties were invited to serve as facilitators for the session. One facilitator was assigned to each group of four to six students, without the requirement to be in the same specialty. Prior to the session, facilitators were sent guidance for the session via email (Appendix F), as

Copyright © 2024 Jamison et al. This is an open-access publication distributed under the terms of the Creative Commons Attribution license.

well as the 6-minute video (Appendix B) and evaluation rubric (Appendix D) sent to participants. Overall, the materials provided for facilitators required about 15 minutes of review prior to the 90-minute session. There were no requirements for serving as a facilitator beyond level of training, reviewing the provided course materials, and expressing interest in the session.

Session Logistics

In 2021, each group of participants and their facilitator were assigned a unique virtual meeting room (using Zoom) for the session; in 2022, each group met in a room on campus equipped with a whiteboard and dry erase markers. During the session, each participant had 15 minutes to deliver their MCT (up to 6 minutes) and then to receive verbal feedback from their peers (about 9 minutes) moderated by the facilitator. Students and facilitators were encouraged to describe aspects of the MCT that they found effective as well as those that might be improved. During the session, the facilitator filled out a formative evaluation rubric (Appendix D) for each participant, which was emailed to the student and the session organizer after the workshop. At the conclusion of each participant's MCT, they were prompted to reflect briefly on the experience of delivering the talk and receiving feedback and to share one key insight they had learned about teaching within the given topic and format. We include here a sample chalk talk given by a participant for reference (Appendix G [author owned and not copyrighted]), though this video was not provided to participants.

Evaluation

Each year, participants completed a required electronic presession survey (Appendix A) as part of the session sign-up form and were invited to fill out an optional deidentified electronic postsession survey (Appendix H) immediately after the session. Both surveys had items based on the learning objectives of the MCT session and assessing students' prior experience with MCTs. Items in the presurvey utilized a 5-point Likert scale (1 = *not at all well,* 5 = *extremely well*) and were reviewed for clarity, content validity, and accuracy by two expert faculty members using a think-aloud process. Students provided both quantitative and qualitative feedback on the module in the postsession survey. Three questions in the postsession survey mirrored those in the presession survey; two additional questions were included about the knowledge students had gained from the session. Two open-ended questions asked students what they liked best about the session and how the session could be improved. Facilitators were asked to send completed MCT evaluation rubrics to the course director as a measure of students' achievement of the learning objectives.

For statistical analysis of Likert-scale responses, mean response values to corresponding questions were compared using *t* tests assuming unequal variance. Qualitative survey responses were subjected to inductive thematic analysis.

Results

Over the 2 years that the workshop has been offered, 26% of fourth-year medical students (95 of 370) opted to participate, 28% (57 of 200) in 2021 and 22% (38 of 170) in 2022. Students entering 16 different specialties took part in the workshop, with a majority of participants entering internal medicine, pediatrics, or internal medicine-pediatrics (Table 1). To maintain optimal group sizes and student-facilitator ratios, 12 facilitators were recruited in 2021, and seven facilitators were recruited in 2022. Overall, 79% of facilitators (15 of 19) were internal medicine faculty or residents, and 21% (four of 19) were pediatrics faculty or residents. Sixty-eight percent of facilitators (13 of 19) were senior residents, with the remainder being faculty (Table 2). Most resident facilitators were participants in their programs' resident-educator organizations.

In 2021, 91% of students (52 of 57) responded to the postsession survey, and in 2022, 66% of students (25 of 38) responded to it, for a total of 81% of students (77 of 95). The difference in response rates between the 2 years was most likely due to the change in session format, as the virtual format in 2021 allowed students to easily complete the evaluation electronically prior to leaving the session. The pre- and postsession surveys shared common questions related to students' confidence describing strategies to deliver effective MCTs and delivering

Table 1. Workshop Participants by Specialty

	No. (%)		
**Specialty**	**2021 (*N* = 57)**	**2022 (*N* = 38)**	**Total (*N* = 95)**
Internal medicine	14 (25)	15 (40)	29 (30)
Pediatrics	9 (16)	6 (16)	15 (16)
Internal medicine-pediatrics	3 (5)	2 (5)	5 (5)
General surgery	5 (9)	2 (5)	7 (7)
Surgical subspecialty	2 (4)	0 (0)	2 (2)
Obstetrics/gynecology	2 (4)	4 (10)	6 (6)
Emergency medicine	8 (14)	1 (3)	9 (10)
Anesthesiology	2 (4)	0 (0)	2 (2)
Neurology	4 (7)	1 (3)	5 (5)
Psychiatry	4 (7)	2 (5)	6 (6)
Family medicine	1 (2)	2 (5)	3 (3)
Radiology	1 (2)	1 (3)	2 (2)
Dermatology	1 (2)	0 (0)	1 (1)
Ear, nose, and throat	0(0)	1 (3)	1 (1)
Physical medicine and rehabilitation	0 (0)	1 (3)	1 (1)
Pediatrics-genetics	1 (2)	0 (0)	1 (1)

Copyright © 2024 Jamison et al. This is an open-access publication distributed under the terms of the Creative Commons Attribution license.

Table 2. Session Facilitator Characteristics

Professional Characteristics	No. (%)		
	2021 (*N* = 12)	2022 (*N* = 7)	Total (*N* = 19)
Internal medicine	8 (67)	7 (100)	15 (79)
Pediatrics	4 (33)	0 (0)	4 (21)
Resident	9 (75)	4 (57)	13 (68)
Faculty	3 (25)	3 (43)	6 (32)

an MCT, both of which showed significant improvements on the postsession survey (Table 3). Students' self-reported number of previously performed MCTs in their repertoire increased from a median of one MCT prior to the workshop to two to three MCTs after the workshop. The postsession survey indicated that students found the session valuable for improving their teaching skills and gaining clinical knowledge (Table 3).

Students' qualitative feedback on the postsession survey revealed the value of receiving feedback on their teaching (31 of 77 respondents, 40%), the benefits of practicing teaching skills in a safe environment (21 of 77, 27%), the usefulness of experiencing other students' MTs (13 of 77, 17%), and the advantage of small groups with students in the same or similar specialties (eight of 77, 10%; Table 4). The most-described opportunity for improvement for the workshop was the desire for a repository of all students' MCTs that could be used after the session. While the feedback from the workshop in 2021 was favorable, 10% of students (five of 52) specifically noted that they would have preferred the session occurred in person.

Qualitative analysis of the formative evaluation rubrics filled out by facilitators yielded several insights. Facilitators frequently praised the students' ability to distill complex clinical topics into short MCTs with helpful frameworks. Facilitators commented positively on the relevance of topics chosen by students, even in groups with multiple specialties represented (e.g., a future otolaryngologist delivering an MCT about tracheostomy care for nonotolaryngologists). Common opportunities for student improvement noted by facilitators included crafting more precise learning objectives, stating learning objectives more clearly, narrowing the focus of the talk, incorporating more checks for understanding, and reducing the length of the MCT (many exceeded the recommended 6-minute limit).

Discussion

The structure and flexibility of medical school capstone courses offer an ideal opportunity for better preparing students for their teaching roles as interns. We successfully demonstrated the implementation of an MCT session in a resource- and time-efficient manner. Our session required minimal time commitment from facilitators and was accommodated within the curriculum's time constraints. While creating an MCT is challenging and requires preparation, students found value in the practice and feedback, as well as reporting increased confidence in their abilities as teachers. Applying educational principles in creating and delivering an MCT may foster a deeper understanding of those principles compared to a lecture-based session, highlighting another benefit of the flipped classroom format.

One of the strengths of the workshop is its versatility. The workshop has been delivered successfully and evaluated positively in both the virtual and in-person formats. Additionally, the positive reception by a broad range of future specialties suggests that the teaching objectives of the session are broadly relevant. The flexible format and diversity of potential participants contribute to the workshop's potential replicability in other settings.

Another strength of this workshop relates to the value of sharing MCTs among students in the same or similar specialties. By viewing their peers' talks, students reported learning clinical information related to their future specialty, as well as gaining

Table 3. Quantitative Survey Results

Survey Question	2021			2022			Total		
	M (*SD*)			*M* (*SD*)			*M* (*SD*)		
	Presurvey (*N* = 57)	Postsurvey (*N* = 52)	*p*	Presurvey (*N* = 38)	Postsurvey (*N* = 25)	*p*	Presurvey (*N* = 95)	Postsurvey (*N* = 77)	*p*
How well can you describe strategies for effectively delivering a mini chalk talk?[a]	2.1 (0.7)	4.1 (0.6)	<.01	2.2 (0.8)	4.1 (0.6)	<.01	2.1 (0.8)	4.1 (0.6)	<.01
How confident do you feel in your ability to give an effective mini chalk talk?[a]	2.4 (0.7)	3.8 (0.6)	<.01	2.5 (0.9)	4.1 (0.6)	<.01	2.4 (0.8)	3.9 (0.6)	<01
How much clinical content did you learn from your peers?[b]		4.3 (0.8)			4.6 (0.7)			4.4 (0.8)	
How much teaching style or technique did you learn from your peers?[b]		4.4 (0.8)			4.8 (0.4)			4.5 (0.7)	

[a]Rated on a 5-point Likert scale (1 = *not at all*, 5 = *extremely*).
[b]Rated on a 5-point Likert scale (1 = *did not learn*, 5 = *a great amount*).

Copyright © 2024 Jamison et al. This is an open-access publication distributed under the terms of the Creative Commons Attribution license.

Table 4. Qualitative Results With Sample Responses

Theme	No. (%)			Sample Response
	2021 ($N = 52$)	2022 ($N = 25$)	Total ($N = 77$)	
Value of receiving feedback from the session	19 (36)	12 (48)	31 (40)	"Being in a small group and getting feedback from peers and facilitator was very useful."
Benefits of practicing teaching skills in a safe environment	13 (25)	8 (32)	21 (27)	"I loved the fact that we got to make our own chalk talks and share. I thought it was an excellent time to safely practice our skills with peers."
Usefulness of hearing other students' talks	9 (17)	4 (16)	13 (17)	"Great seeing other people's chalk talks and getting ideas about other ways to make chalk talks for the future."
Advantage of small groups with peers in the same/similar specialties	6 (12)	2 (8)	8 (10)	"Getting to practice our talk among our specialty-mates was helpful since they are the audience we will be among!"
Value added to session by facilitators	4 (8)	4 (16)	8 (10)	"Dr. X was a fantastic facilitator—appreciated her frank feedback."
Effectiveness of presession resources	3 (6)	0 (0)	3 (4)	"Very effective, having the videos with the tips before the presentations was very useful."
Desire for greater training on delivering feedback	3 (6)	0 (0)	3 (4)	"I wasn't great at giving feedback, especially for the first few chalk talks. I wonder if it might benefit from a pre-small group session where we watch a few sample chalk talks, on the same topic, with different approaches and levels of quality."
Request for repository of students' talks	4 (8)	3 (12)	7 (9)	"Have each student, if possible, bring their prep sheet and print out enough to share with other students so they can implement into their repertoire."
Preference for in-person session	5 (10)			"It would be better/more effective in person, but could not be avoided this year!"

exposure to other teaching styles. As one student noted, specialty alignment within groups also added value to the feedback received, as their audience for the session was similar to that of their future educational setting. Interestingly, despite the limited number of specialties represented among facilitators, participant comments related to the facilitators were overwhelmingly positive and focused on the value of feedback that they delivered, suggesting that a commitment to effective teaching practices is more important than specialty alignment for facilitators. Thus, we believe our results suggest that other institutions replicating this intervention should focus on recruiting faculty and resident facilitators with an interest in medical education regardless of specialty.

One opportunity for improvement highlighted by students in the postsession survey is the suggestion for a centralized collection of all presented MCTs that students could access and utilize to enhance their own skill set. To achieve this objective, students could be asked to submit their teaching script and a completed board image for in-person MCTs or a recording for virtual MCTs. These deliverables could then be added to a file-sharing system and sent to students.

While the MCT session yielded positive outcomes, there are limitations to consider. First, differences among facilitators and student groups likely led to variable experiences of individual participants, especially with respect to feedback on MCTs. Providing effective feedback is a common goal among fourth-year students,[22] and sharing a framework for effective feedback[23] prior to the session could bolster the development of this skill during the session. Providing instruction to facilitators on a standardized approach for delivering verbal feedback could be a potential solution, though it could add to preparation or session time. Conducting a concise training session for facilitators on instructing students in crafting effective learning objectives and delivering impactful feedback could enhance facilitators' proficiency in this domain and standardize learners' experience. Additionally, the workshop evaluation strategies we used, focusing on learner-reported knowledge and confidence, may fail to correlate with objective measures of teaching skills or knowledge. An approach for assessing higher-level outcomes might be to leverage facilitator evaluations of MCTs. Comparing the virtual and in-person iterations of our educational intervention poses challenges. However, the positive feedback from both suggests promising prospects for future implementation of either format.

In conclusion, students found our MCT workshop to be a beneficial setting in which to develop their teaching skills and receive feedback from both peers and a facilitator. Since the session can be incorporated into a preexisting capstone course with minimal additional in-class time, it can be replicated in a variety of curricular contexts to facilitate students' transition to their role as educators.

Appendices

A. Presurvey Questions.docx

B. How to Prepare an Effective Mini Chalk Talk Video.mp4

Copyright © 2024 Jamison et al. This is an open-access publication distributed under the terms of the Creative Commons Attribution license.

C. Mini Chalk Talk Tip Sheet.docx

D. Mini Chalk Talk Observation Form.docx

E. Mini Chalk Talk Preparation Worksheet.docx

F. Facilitator Email.docx

G. Sample Mini Chalk Talk.mp4

H. Postsurvey Questions.docx

All appendices are peer reviewed as integral parts of the Original Publication.

Trevor Jamison, MD: Second-Year Internal Medicine Resident, Department of Medicine, Baylor College of Medicine; ORCID: https://orcid.org/0009-0005-4005-5182

Zaven Sargsyan, MD: Associate Professor, Division of General Internal Medicine, Department of Medicine, Baylor College of Medicine

Uma Ayyala, MD: Assistant Professor, Division of Pulmonary, Critical Care, and Sleep Medicine, Department of Medicine, Baylor College of Medicine

Stephanie Sherman, MD: Associate Professor, Division of General Internal Medicine, Department of Medicine, Baylor College of Medicine

Holland Kaplan, MD: Assistant Professor, Division of General Internal Medicine, Department of Medicine, and Center for Medical Ethics and Health Policy, Baylor College of Medicine

Disclosures
None to report.

Funding/Support
None to report.

Ethical Approval
Reported as not applicable.

References

1. Dandavino M, Snell M, Wiseman J. Why medical students should learn how to teach. *Med Teach*. 2007;29(6):558-565. https://doi.org/10.1080/01421590701477449
2. Sherman S, Kumar S, Lim J, Sargsyan Z, Ratan B, Turner T. Measuring teacher identity during the transition from medical school to residency. *Proc (Bayl Univ Med Cent)*. 2020;33(4): 566-571. https://doi.org/10.1080/08998280.2020.1792747
3. Hill AG, Srinivasa S, Hawken SJ, et al. Impact of a resident-as-teacher workshop on teaching behavior of interns and learning outcomes of medical students. *J Grad Med Educ*. 2012;4(1):34-41. https://doi.org/10.4300/JGME-D-11-00062.1
4. Donovan AK, Linz DR, Rubio DM, McNeil MA, Spagnoletti CL. Teaching to teach: an effective and feasible teaching curriculum for internal medicine residents. *South Med J*. 2018;111(12): 733-738. https://doi.org/10.14423/SMJ.0000000000000908
5. Pasquinelli L, Greenberg L. A review of medical school programs that train medical students as teachers (MED-SATS). *Teach Learn Med*. 2008;20(1):73-81. https://doi.org/10.1080/10401330701798337
6. Soriano RP, Blatt B, Coplit L, et al. Teaching medical students how to teach: a national survey of medical students-as-teachers programs in U.S. medical schools. *Acad Med*. 2010;85(11): 1725-1731. https://doi.org/10.1097/ACM.0b013e3181f53273
7. Yu TC, Wilson NC, Singh PP, Lemanu DP, Hawken SJ, Hill AG. Medical students-as-teachers: a systematic review of peer-assisted teaching during medical school. *Adv Med Educ Pract*. 2011;2:157-172. https://doi.org/10.2147/AMEP.S14383
8. Marton GE, McCullough B, Ramnanan CJ. A review of teaching skills development programmes for medical students. *Med Educ*. 2015;49(2):149-160. https://doi.org/10.1111/medu.12571
9. Gainor J, Patel NK, George PF, Macnamara MMC, Dollase RH, Taylor JS. An intensive medical education elective for senior medical students. *R I Med J (2013)*. 2014;97(7):40-44.
10. Yoon MH, Blatt BC, Greenberg LW. Medical students' professional development as educators revealed through reflections on their teaching following a students-as-teachers course. *Teach Learn Med*. 2017;29(4):411-419. https://doi.org/10.1080/10401334.2017.1302801
11. Fuchs MA, Schwartz AW, Caton JB, Gooding H, Richards JB. Defining student-as-teacher curricula in the absence of national guidelines: an innovative model. *Acad Med*. 2022;97(6):832-838. https://doi.org/10.1097/ACM.0000000000004589
12. Cohen A, Steinert Y, Ruano Cea E. Teaching medical students to teach: a narrative review and literature-informed recommendations for student-as-teacher curricula. *Acad Med*. 2022;97(6):909-922. https://doi.org/10.1097/ACM.0000000000004608
13. Germann CA, Strout TD, Park YS, Tekian A. Senior-year curriculum in U.S. medical schools: a scoping review. *Teach Learn Med*. 2020;32(1):34-44. https://doi.org/10.1080/10401334.2019.1618307
14. Cosgrove EM, Ryan MJ, Wenrich MD. Empowering fourth-year students: the value of the senior year. *Acad Med*. 2014;89(4): 533-535. https://doi.org/10.1097/ACM.0000000000000191
15. Elnicki DM, Gallagher S, Willet L, et al; Clerkship Directors in Internal Medicine/Association of Program Directors in Internal Medicine Committee on Transition to Internship. Course offerings in the fourth year of medical school: how U.S. medical schools are preparing students for internship. *Acad Med*. 2015;90(10): 1324-1330. https://doi.org/10.1097/ACM.0000000000000796
16. Singh N, Phoon CKL. Not yet a dinosaur: the chalk talk. *Adv Physiol Educ*. 2021;45(1):61-66. https://doi.org/10.1152/advan.00126.2020

Copyright © 2024 Jamison et al. This is an open-access publication distributed under the terms of the Creative Commons Attribution license.

17. Pitt MB, Orlander JD. Bringing mini-chalk talks to the bedside to enhance clinical teaching. *Med Educ Online*. 2017;22(1): 1264120. https://doi.org/10.1080/10872981.2017.1264120
18. Morris GA, Vaz K, Russo J, et al. Implementing mini-chalk talks to enhance teaching and learning. *Clin Teach*. 2021;18(6):577-582. https://doi.org/10.1111/tct.13394
19. Palamara K, Ray A. Resident-as-teacher workshop. *MedEdPORTAL*. 2014;10:9673. https://doi.org/10.15766/mep_2374-8265.9673
20. Zackoff M, Jerardi K, Unaka N, Fleming A, Patterson B, Klein M. Resident-as-teacher curriculum and assessment tool for brief didactic teaching in pediatrics. *MedEdPORTAL*. 2015;11:10030. https://doi.org/10.15766/mep_2374-8265.10030
21. Lescinskas E, Sargsyan Z, Ayyala US, Fisher J. Preparing for medical internship: a case-based strategy to teach management of common overnight calls to students. *MedEdPORTAL*. 2020;16: 10966. https://doi.org/10.15766/mep_2374-8265.10966
22. Hughes JDM, Azzi E, Rose GW, Ramnanan CJ, Khamisa K. A survey of senior medical students' attitudes and awareness toward teaching and participation in a formal clinical teaching elective: a Canadian perspective. *Med Educ Online*. 2017;22(1): 1270022. https://doi.org/10.1080/10872981.2016.1270022
23. Hall C, Peleva E, Vithlani RH, et al. FEEDBK: a novel approach for providing feedback. *Clin Teach*. 2020;17(1):76-80. https://doi.org/10.1111/tct.13026

Received: October 16, 2023
Accepted: February 26, 2024
Published: June 25, 2024

Copyright © 2024 Jamison et al. This is an open-access publication distributed under the terms of the Creative Commons Attribution license.
